# What are the perspectives of patients with hand and wrist conditions, chronic pain, and patients recovering from stroke on the use of patient and outcome information in everyday care? A Mixed-Methods study

**DOI:** 10.1007/s11136-024-03685-1

**Published:** 2024-06-05

**Authors:** Grada R. Arends, Nina L. Loos, Yara E. van Kooij, Kasia Tabeau, Willemijn A. de Ridder, Ruud W. Selles, Joris Veltkamp, Outcome-Based Healthcare Research Group, Robbert M. Wouters

**Affiliations:** 1 Department of Rehabilitation Medicine, Erasmus MC, Rotterdam, The Netherlands; 2 Department of Plastic, Reconstructive, and Hand Surgery, Erasmus MC, Room EE-1589, PO Box 2040, 3000 CA Rotterdam, The Netherlands; 3 Xpert Handtherapie, Utrecht, The Netherlands; 4 Erasmus School of Health Policy and Management, Erasmus University Rotterdam, Rotterdam, The Netherlands

**Keywords:** Patient-centered care, Patient-reported outcome measures, Value-based healthcare, Decision support

## Abstract

**Purpose:**

To evaluate the patients’ perspectives on the use of patient- and outcome information tools in everyday care and to investigate which characteristics affect general understanding and perceived value of patient- and outcome information.

**Methods:**

This mixed-methods study included surveys and interviews on understanding, experience, decision-support, and perceived value in patients with hand and wrist conditions and chronic pain. We synthesized our quantitative and qualitative findings using a triangulation protocol and identified factors independently associated with general understanding and perceived value of patient- and outcome information using hierarchical logistic regression.

**Results:**

We included 3379 patients. The data triangulation indicated that patients understand the outcome information, they find it valuable, it supports decision-making, and it improves patient-clinician interaction. The following variables were independently associated with better general understanding: having more difficulty with questionnaires (standardized odds ratio 0.34 [95%-CI 031–0.38]), having a finger condition (0.72 [0.57–0.92]), longer follow-up (0.75 [0.61–0.91]), and undergoing surgical treatment (ref: non-surgical treatment, 1.33 [1.11–1.59]). For more general value, these were: having more difficulty with questionnaires (0.40 [0.36–0.44]), having a wrist condition (0.71 [0.54–0.92]), better hand function (1.12 [1.02–1.22]), and requiring help with questionnaires (1.65 [1.33–2.05]).

**Conclusion:**

Patients value the use of patient- and outcome information tools in daily care and find it easy to understand. The factors associated with understanding and value can be targeted to personalized and value-based healthcare. We recommend using outcome information to improve patient independence, empowerment, and involvement in decision-making.

**Supplementary Information:**

The online version contains supplementary material available at 10.1007/s11136-024-03685-1.

## Plain English summary

**1. Why is this study needed?**

It is unknown how patients experience the use of outcome information tools by their clinicians. These tools might help patients by increasing their understanding of the information provided and by creating decision-support.

**2. What is the key problem/issue/question this manuscript addresses?**

This manuscript investigates what patients think about the use of patient- and outcome information tools. It is also needed to investigate what influences patient’s general understanding and perceived value of patient- and outcome information.

**3. What is the main point of your study?**

Patients value the use of patient- and outcome information tools in daily care and find it easy to understand.

**4. What are your main results and what do they mean?**

Our main results are that patients value outcome information tools and find them easy to understand. When clinicians use outcome information, they can improve patient independence, empowerment, and involvement in decision-making.

## Introduction

Value-based healthcare (VBHC) strives to deliver the highest possible quality of care against reasonable costs, measured with patient-relevant outcomes [1]. In the transition to VBHC, healthcare organizations are increasingly collecting outcome information [2, 3], where outcome information refers to outcomes collected through patient-reported (PROMs) or clinician-reported outcome measures (CROs). Using outcome information improves patient-clinician communication, supports patients in discussing issues and symptoms [4–6], and facilitates patients in monitoring their symptoms and understanding their health status [4, 5]. Outcome information also supports decisions for both clinicians and patients, improving patient-centered healthcare and improving treatment outcomes, ultimately improving quality of life [2]. However, implementing outcome information in daily clinical practice is challenging [7, 8].

To facilitate implementation, effective translation of outcome information into daily clinical care is important [1, 9]. Technology can play an important role in this process. For example, dashboards with intuitive outcome information tools (OITs) can help clinicians integrate outcome information into their everyday workflow [10]. Using OITs improves patients’ insight into their symptoms and health status, treatment expectations, and shared decision-making [11–15]. However, previously studied OITs did not use individual patient data but group means, and these tools were not digital.

We developed and implemented several OITs for clinicians based on individual PROMs and CROs data. It is unknown how patients experience the use of such OITs by clinicians. Integrating outcome information in daily care through OITs may empower patients by increasing their understanding of the information provided and creating decision-support. Although the OITs were designed to personalize healthcare, hypothetically, patients may not understand them or perceive their use as less personal, as clinicians may use their computers more frequently. Thus, more knowledge of patient experiences with clinicians’ use of OITs is required.

This study investigated how well patients understand and experience patient and outcome information through OITs, how valuable they think using this information is, and if it provides decision-support. Also, we investigated which patient and treatment characteristics affect the patients’ general understanding and perceived value of outcome information through OITs. To increase generalizability, we studied the perspectives of different patient populations: hand- and wrist conditions, chronic pain, and stroke rehabilitation. We chose these as they have different quality of life, receive both elective and non-elective care, and strongly differ in burden of care and limitations in daily living [16, 17].

## Methods

### Study design

This mixed-methods study, comprising patient surveys and semi-structured interviews, is reported according to the Mixed Methods Article Reporting Standards (MMARS) [18]. The Medical Ethics Committee of Erasmus MC approved this study (MEC-2022-0286). All participants provided informed consent.

### Setting

This study was a collaboration between three different organizations: (1) a specialized clinic for hand and wrist care (Xpert Clinics), (2) a rehabilitation center (Rijndam Rehabilitation), and (3) a general hospital [Onze Lieve Vrouwe Gasthuis (OLVG)\.

### Patient and outcome information tools (OITs)

We developed six OITs based on individual PROMs and CROs data together with clinicians and patients aiming to improve the clinicians’ use of outcome information and support decision-making and treatment progress monitoring. These include (1) visuals of patient information, (2) visuals of outcome information, (3) the personal request for help and treatment goals, (4) screening tools for pain, function, and mental health, (5) individual predictions of recovery and treatment effect, and (6) identification and feedback of 'extreme values' with color coding. The OITs are incorporated into dashboards accessible via electronic patient records (Supplementary Figure 1A-C), except for OLVG, where a prediction model is used as a stand-alone OIT (Supplementary Figure 1D). All clinicians were trained on the use and interpretation of the OITs, but although usage is encouraged, actual usage varies and depends on individual preference. The OITs used in this study are described below.

#### Visuals of patient information

The dashboard provides individual patient information on health status, including medical history and sociodemographic characteristics.

#### Personal request for help and treatment goals

These are collected before the first clinician consultation using the Patient Specific Needs Evaluation [19]; a short patient-reported questionnaire.

#### Screening tools for pain, hand function, and mental health

We collect short, patient-reported questionnaires on these domains before the first clinician consultation. These existing tools include three 0–10 Numeric Rating Scales (NRSs) for pain and hand function and the 4-item Ultra-short Mental Health Screening Tool [20].

#### Individual predictions

At Xpert Clinics, the prediction models make individual predictions on the probability of improving by at least two points on the NRS pain and hand function after treatment [21]. At OLVG, the models predict the improvement of upper extremity function over time following stroke [22].

#### Visuals of outcome information

The dashboards visualize PROMs and CROs during the treatment course using graphs and tables, including individual patient data against norm data and the expected time to return to work (Supplementary Figure 1A, C-D).

#### Identification and feedback of extreme values

Extreme values are values that deviate from the expected, based on patient data. Examples include values with little room for improvement (e.g., the highest possible function score at baseline) or risk factors for poor recovery (e.g., pain catastrophizing behavior). Extreme values are marked using color coding (i.e., green, orange, red) and can occur in outcome information and screening tools.

### Participants

Between April and July 2022, we invited adult patients with an intake or three-month follow-up appointment at Xpert Clinics or an intake appointment at Rijndam to complete our survey. For the descriptive analysis, patients with complete data on our survey and sociodemographics were included.

For the hierarchical regression analysis on general understanding and perceived value, we included patients with complete data on the survey at intake and other regression variables, i.e., sociodemographics, mental health factors, and treatment factors. Patients with missing data were excluded from the regression analysis.

We conducted semi-structured interviews with adult patients from all participating organizations. We used purposive sampling with maximum variation to include a variety of patients with different ages, genders, socioeconomic backgrounds, and (health) literacy levels [23–27]. We excluded patients who did not speak B1 level Dutch.

### Surveys

The survey was developed in conjunction with clinicians and patients and consisted of questions on the patient’s understanding of and their experience with the use of OITs, their perceived value of OITs, and whether they provided decision-support for their treatment. These aspects were rated separately per OIT. Questions on each OIT were only asked if patients indicated in the survey that they believed the respective OIT was used during the consultation. The question on decision-support was not asked for the visuals of outcome information since this OIT is not available at intake, and therefore it cannot support decision-making on the initial treatment. Similarly, the question on understanding was not asked for the personal request for help and individual treatment goals and the question on decision-support not for the personal request for help, because patients provide this information themselves. If patients indicated that their clinician did not use a specific OIT, they were asked whether they would have liked to discuss that one.

The survey also contained two screening questions on (health) literacy: (1) “*Many people find it difficult to properly understand and complete a questionnaire, how is this for you?*” (0–10, with 0 indicating always and 10 indicating never) and (2) “*Does anyone ever help you completing questionnaires or reading letters*?” (Yes/No)[28].

Our primary outcomes were two general questions on the patients’ understanding and perceived value of the OITs; “*In general, did you understand the use of the discussed OITs?”* (0–10, 10 = completely) and *“In general, how valuable do you think the use of the discussed OITs is?”* (0–10, 10 = very valuable). The other survey items were secondary outcomes.

### Interviews

We developed an interview guide with topics on each different OIT. After developing the guide, we conducted a test-interview, followed by a cognitive debriefing to identify points for improvement in the interview guide. We adapted the interview guide to the available OITs in each organization. The interviewer showed examples of the OITs during the interview.

ID interviewed the patient from OLVG and GRA conducted all other interviews. Interviews were conducted in Dutch and lasted approximately 45 min. We performed interviews until no new information emerged, indicating data saturation was reached [29, 30].

### Additional measurements

Additional data were collected from patients treated at Xpert Clinics as part of routine care to be used as input for the hierarchical logistic regression. We created four categories of variables: (1) demographic and treatment characteristics, (2) mental health, (3) pain and function, and (4) socioeconomic factors.

Demographic and treatment characteristics included age, sex, whether the appointment was a second opinion, duration of symptoms, medical history, whether the patient’s dominant hand was affected, treatment track, and treatment type (surgical, nonsurgical, or no treatment).

4 Mental health included the aforementioned Ultrashort Mental Health Screening Tool, which is a valid and reliable measure for illness perceptions, psychological distress, and pain catastrophizing [20].

Pain in rest, pain during loading, and hand function were measured using a 0–10 Numeric Rating Scale (higher scores indicating more pain but better hand function) [31].

Socioeconomic factors included questions on limited (health) literacy from the survey and a socioeconomic status (SES) score based on postal code. The SES-score was obtained from the Dutch Central Bureau for Statistics (CBS) and calculated based on wealth (disposable income, capital, and household composition), educational level, and recent occupational history [32]. The SES-score measures how wealthy or socially advantaged a specific postal area is compared to other postal areas. The SES-score ranges from approximately − 2.0 to 1.0 with higher scores indicating a higher socioeconomic status.

### Data analysis

#### Primary objective

We performed descriptive statistics on the survey questions using the median score with interquartile range.

The interviews were audio-recorded and transcribed verbatim. We analyzed the interviews using qualitative content analysis with inductive coding to identify shared themes using MAXQDA [23, 24, 33, 34]. Two authors (GRA and YvK) open-coded the interviews separately. After the fifth interview, we adapted the interview guide. We member-checked the results by discussing the main outcomes with each participant and discussed the results within the research group.

We followed a triangulation protocol to combine our quantitative and qualitative findings [35]. The first step involved organizing the data following the components of our first research aim: (1) patients’ understanding of the OITs, (2) patients’ perceived value of the OITs, and (3) if the OITs provided patients with decision-support. We then performed convergent coding, which included comparing the results from the surveys and the interviews separately to find initial answers to our research questions based on that data collection method. In the third step, we performed a convergence assessment by discussing the results from the previous steps multiple times with the research team. Finally, we conducted a comprehensive comparison, combining all findings from both the survey and interviews.

#### Secondary objective

Large ceiling effects were present in our data for the questions on general understanding and general perceived value of the OITs (Supplementary Figure 2A-B), which would have made the results of linear regression unreliable [36]. We, therefore, dichotomized the questions on general understanding and perceived value of the outcome information. Patients scoring below the median on understanding were dichotomized as “worse understanding”, while patients scoring the median or higher as “better understanding”. Similarly, patients scoring below the median on perceived value were dichotomized as “less perceived value”, while patients scoring the median or higher as “more perceived value”. Before analysis, the question on (health) literacy: “*Many people find it difficult to properly understand and complete a questionnaire, how is this for you?*” was converted by subtracting the patients’ raw scores from the maximal score of 10, so that a score of 0 indicated “never” and a score of 10 indicated “always”.

To investigate the association of patient characteristics with the patients’ overall understanding and perceived value of using outcome information, we performed a hierarchical logistic regression analysis. In this analysis, variables are added in steps to assess if they account for significant variability in the outcome while correcting for other variables. In the first step, the demographic and treatment factors were added; in the second step, mental health; in the third step, pain and function; and in the fourth step, socioeconomic factors. We determined standardized odds ratios to make between-variable comparisons easier and to assess the relative association of each independent variable. To assess the goodness of fit of the different models, we determined the area under the curve (AUC) at each step. We considered an AUC below 0.70 as suboptimal, an AUC between 0.70 and 0.79 as good, and an AUC equal or above 0.80 as excellent [37]. We assessed the relationship between the two questions on (health) literacy using the rank-biserial correlation for comparing dichotomous and ordinal data [38].

All quantitative analyses were performed using R statistical programming, version 4.2.0 [39]. Because we assessed two primary outcome measures, we applied a Bonferonni correction and considered a p-value <0.025 significant.

## Results

**Fig. 1:**
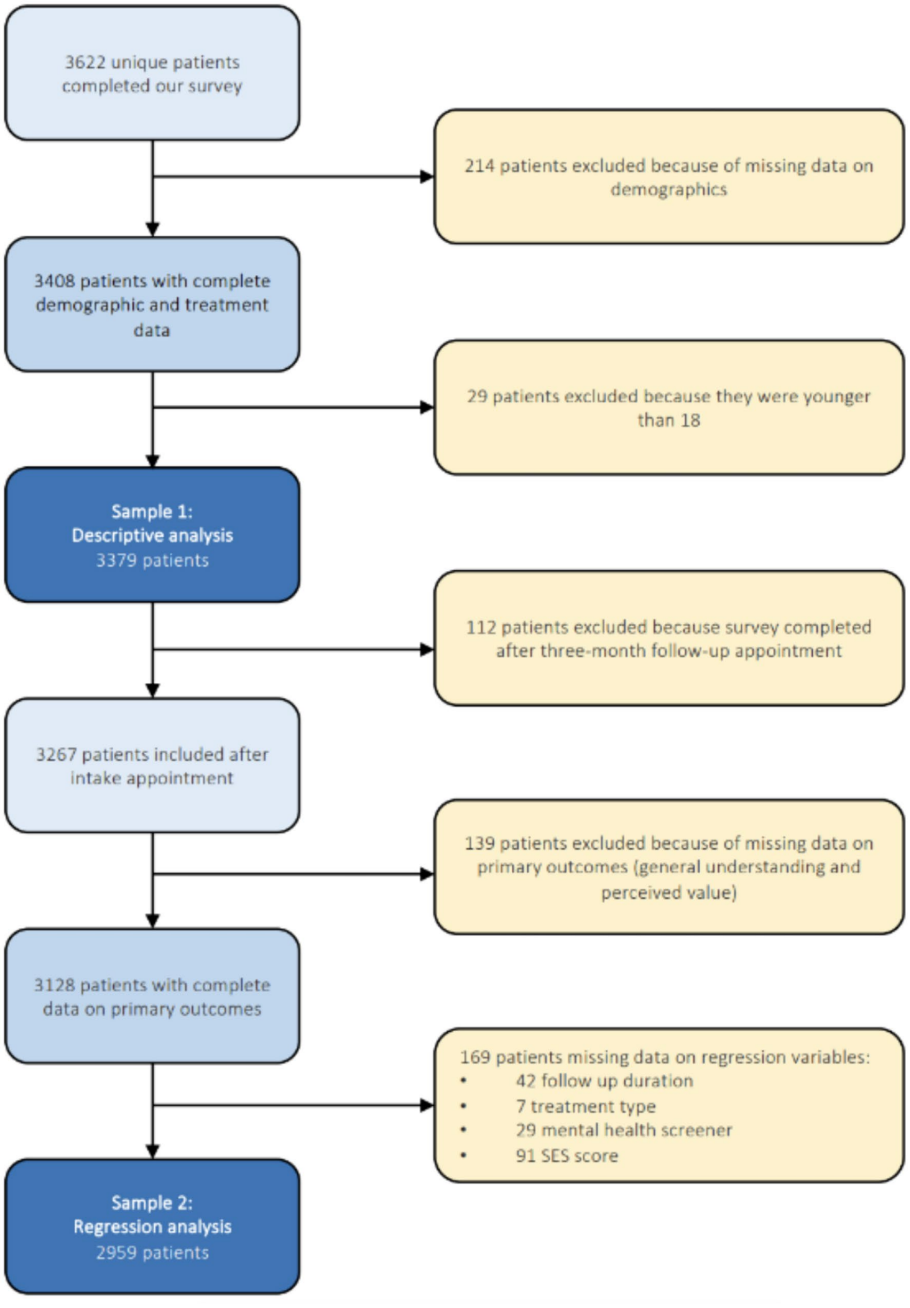
Flowchart of patient selection

We included 3379 patients in the descriptive analyses of the survey and 2959 patients in our logistic regression analysis (Fig. 1). The vast majority were treated at Xpert Clinics (n = 3372). The survey was completed after intake by 3267 patients (97%) and after follow-up by 112 patients (3%, Table 1). After sixteen interviews we reached data saturation, ten were treated at Xpert Clinics, five at Rijndam Rehabilitation, and one at OLVG (Table 2).

**Table 1:** Characteristics of patients included in the descriptive and regression analyses

Characteristics	Sample 1: descriptive analysis (N = 3379)	Sample 2: regression analysis (N = 2959)
Sex		
Female	2147 (64)	1866 (63)
Male	1231 (36)	1093 (37)
Unknown	1 (0)	0 (0)
Age in years (mean ± SD)	57 ± 15	58 ± 15
Time point = Intake	3267 (97)	2959 (100)
Duration of symptoms in months		
Median [IQR]	7.0 [3.0, 20.0]	7.0 [3.0, 20.0]
Missing	7 (0)^a^	
Second Opinion: Yes	387 (12)	334 (11)
Treatment track		
Thumb	766 (23)	636 (22)
Pain rehabilitation	7 (0)	0 (0)
Wrist	778 (23)	1074 (36)
Finger	1199 (36)	565 (19)
Nerve	629 (19)	684 (23)
Treatment type		
No treatment	166 (5)	148 (5)
Surgery	1662 (49)	1433 (48)
Conservative	1536 (46)	1378 (47)
Missing	15 (0)	0 (0)
Track type		
Extended	1327 (39)	1172 (40)
Regular	2001 (59)	1787 (60)
Missing	51 (2)	0 (0)

Variables are displayed with N (%), unless otherwise specified

^a^The seven patients with missing data on symptom duration were treated at Rijndam Rehabilitation. We did not have access to this data

**Table 2:** Characteristics of patients (N = 16) included in the qualitative analysis

Participant	Institution	SES	Sex	Age	Receiving help completing questionnaires^a^
1	Rijndam	− 0.66	F	44	Yes
2	Rijndam	− 0.42	F	46	No
3	Rijndam	− 0.26	F	49	No
4	Rijndam	− 0.06	F	58	No
5	Rijndam	− 0.13	F	31	No
6	OLVG	0.24	M	80	Yes
7	Xpert Clinics	− 0.06	F	64	Yes
8	Xpert Clinics	0.14	M	27	No
9	Xpert Clinics	− 0.11	F	45	Yes
10	Xpert Clinics	0.14	F	27	No
11	Xpert Clinics	0.34	F	52	Yes
12	Xpert Clinics	− 0.33	F	60	Yes
13	Xpert Clinics	0.36	M	61	No
14	Xpert Clinics	− 0.26	M	27	No
15	Xpert Clinics	− 0.21	M	79	No
16	Xpert Clinics	− 0.18	F	45	No

*SES* Social Economic Status. The SES score ranges from approximately − 2.0 to 1.0 with higher scores indicating a higher socioeconomic status

^a^Receiving help completing questionnaires was measured using a screening question

### Triangulated findings

#### Patients’ understanding of outcome information

**Fig. 2:**
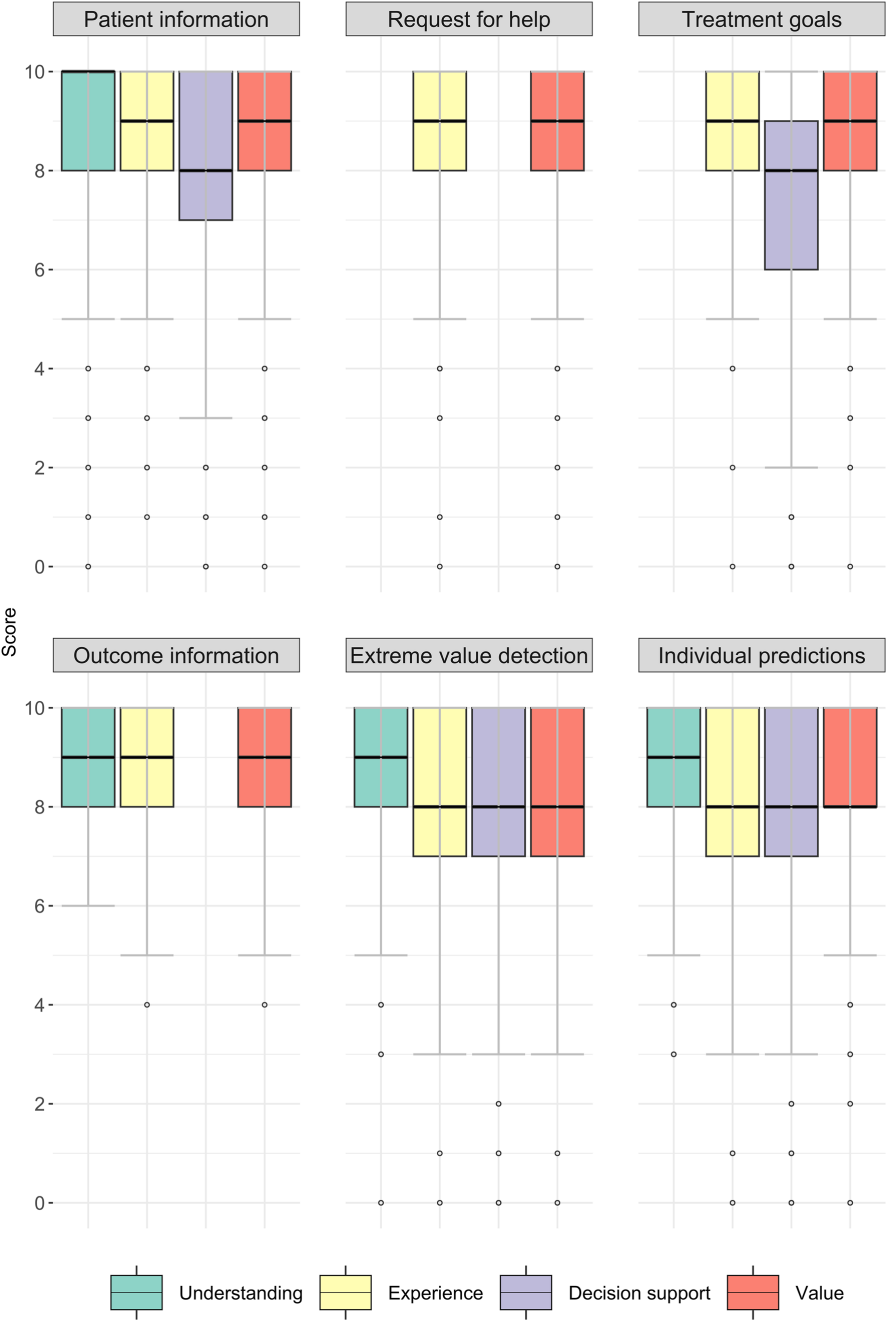
Patients' perspectives on the use of outcome information (n = 3379). In total, 3379 patients completed the survey. However, the number of patient answers differs per question because the questions about understanding, experience, decision-support, and value were only asked if the patient indicated that the OIT was discussed. The figure shows, for each OIT, the patient's answers to the questions: (1) How well did you understand the following OIT (0 = Not at all, 10 = Completely)? (2) How was your experience with the use of the following OIT (0 = Very negative, 10 = Very positive)? (3) To what extent did the following OIT support you in your decision-making about your treatment (0 = To no extent, 10 = To a big extent)? (4) How valuable do you think the use of the following OIT is (0 = Not valuable, 10 = Very valuable)?. Question 1 was not asked for the request for help and the treatment goals and Question 3 not for request for help, as patients complete these themselves. Question 3 was also not asked for the outcome information, as this information is only available after the start of the treatment and, therefore, cannot support the decision-making on the initial treatment choice

The patients’ general understanding of the outcome information was high, with a median of 9 [IQR 8–10] (Table 3). Each separate OIT also scored high on understanding, with a median of 9 or higher (Fig. 2). In the interviews, patients also indicated that the outcome information was clear, especially when the data was visually presented with graphs and different colors (Table 3).

**Table 3:** Triangulation table for the findings of the surveys (N = 3379) and interviews (N = 16)

Research question	Survey questions	Results survey questions (median [IQR])	Conclusion Qualitative analyses	Finding/quote qualitative analyses	Conclusion following synthesis of data
How well do patients understand the applications of patient- and outcome information**?**	Did you understand the use of the discussed applications of outcome information in general (0 = Not all, 10 = Completely)	9 [8–10]	Patients find the data in the applications easy to understand when it is visually presented	“I can see where I stand now and what is possible. I understand it better when it is visually presented.”—Participant 14	The applications are understandable for patients
	How well did you understand the following applications (0 = Not at all, 10 = Completely)?^a^	9 [8–10]			
How valuable do patients find the use of the applications?	How was your experience with the use of the following application (0 = Very negative, 10 = Very positive)?^a^	9 [8–10]	The use of applications helps patients gain understanding of their health state	“You can see that in those three months or that year something has changed. It makes you aware of it.”—Participant 12	Patients find the applications valuable and have a positive experience with the use of the applications
				“We [the patient and clinician] have exactly the same information (…). I think it is easier to adjust [the treatment plan] if you both have the same starting point.”—Participant 2	
	How valuable do you think the use of the discussed applications of outcome information is in general (0 = Not valuable at all, 10 = Very valuable)	8 [7–10]	The use of applications makes patients feel heard	“You get the feeling they have read your file and they are not completely blank”—Participant 10	
	How valuable do you think the use of the following application is (0 = Not valuable, 10 = Very valuable)?^a^	9 [8–10]			
			The use of applications can help motivate patients to complete PROMs and continue treatment	“The more you see you are improving (…) the greater it motivates you to continue.”—Participant 3	
Does the use of applications provide the patient with decision-support?	To what extent did the following application support you in your decision-making about your treatment (0 = To no extent, 10 = To a big extent)?^a^	8 [7–10]	The use of applications supports patient-clinician communication	“I will not say it, they will think ‘here she comes again’. Because of the questionnaire, you cannot get out of it, you’ve already filled it in.”—Participant 11	The applications can help the decision-making process and improve patient-clinician interaction
			The use of applications supports shared decision making	“Of course, this is fantastic! If you can see all of this (…) it would be easy to make a decision, for sure.”—Participant 14	
			The use of applications can empower patients	“There will come a moment that you fall back into your old patterns, (…) This [outcome information] is something you can fall back on. I would like that.”—Participant 3	

^a^Results of survey questions are median [IQR] scores of each question on all applications combine

#### The perceived value of using outcome information

**Fig. 3:**
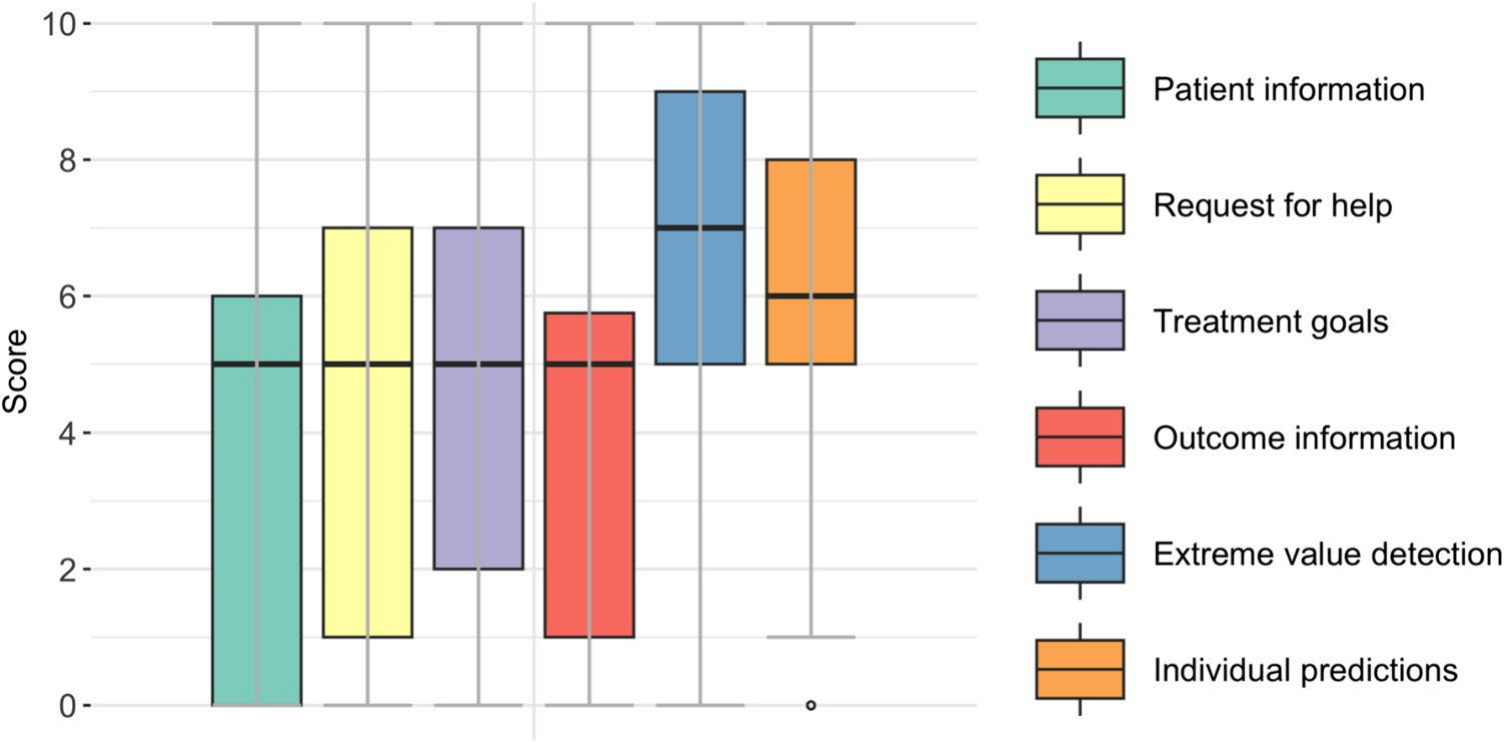
This figure shows answers of patients who indicated the OIT was not discussed during the appointment, on the question: Would you have liked to discuss the following OIT (0 = No, not at all, 10 = Yes, very much)? In total, 3379 patients completed the survey. However, the number of answers differs because the question was only asked if the patient indicated that the OIT was not discussed

The patient’s perceived general value was high, with a median of 8 [IQR 7–10] (Table 3). The visuals of patient information, request for help, treatment goals, and visuals of outcome information were valued with a median of 9, and extreme value detection and individual predictions with a median of 8 (Fig. 2). Patients who indicated certain OITs were not used, scored a median of 5 [IQR from 0–6 to 2–7] on whether they would have liked to discuss patient information, the request for help, treatment goals and outcome information. Similarly, these patients scored a median of 7 [IQR 5–9] and 6 [IQR 5–8] on whether they would have liked to discuss extreme values and individual predictions, respectively (Fig. 3).

In the interviews, patients indicated that they found the outcome information valuable; it improves their understanding of and insight into their health status, symptoms, and treatment progress, motivating them to complete their treatment. Furthermore, using outcome information made them feel heard, and saves time (Table 3). Also, if the clinician discussed outcome information, completing questionnaires became more valuable. Therefore, discussion of outcome information motivated them to complete PROMs (Table 3).

#### Decision-support

All OITs scored a median of 8 on the survey question on decision-support (Fig. 2), and the interviews confirmed these findings (Table 3). Additionally, patients stated that using outcome information enhanced patient-clinician interaction (Table 3). Some participants stated they used the questionnaires to report sensitive issues they find difficult to discuss directly with their clinician. Lastly, using outcome information improved patients' understanding of the information provided by the clinician and managed patients' expectations of treatment effects and recovery (Table 3).

### Factors affecting understanding and perceived value

#### General understanding

**Fig. 4:**
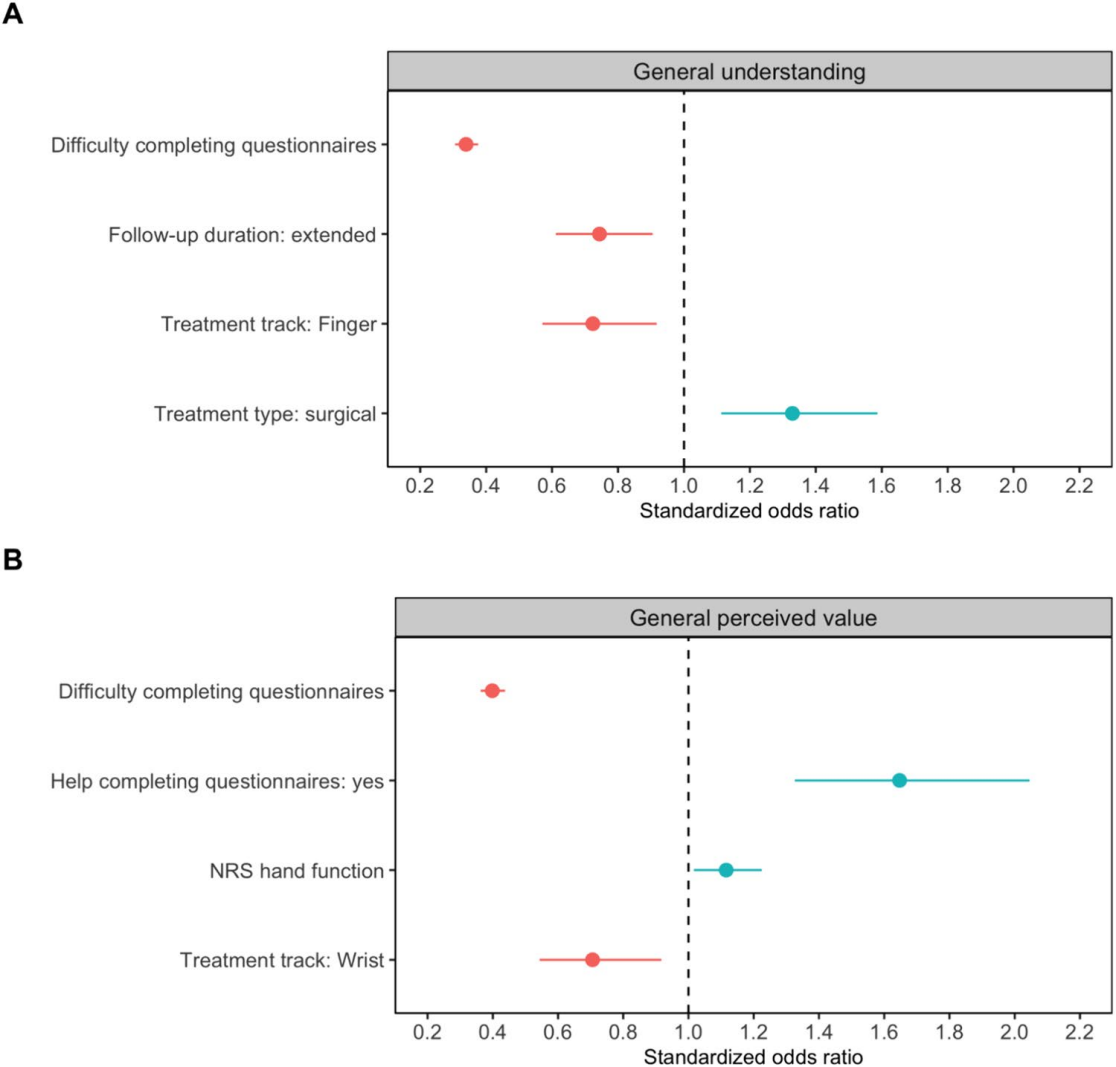
Standardized odds ratios for the final model of the hierarchical logistic regression analysis on the patients’ general understanding (**a**) and general perceived value of outcome information (**b**). The vertical dashed lines indicate no effect. Odds ratios smaller than 1.0 indicate patients have a worse understanding and find outcome information less valuable. Odds ratios larger than 1.0 indicate patients have a better understanding and find outcome information more valuable. General understanding was assessed with the question: Did you understand the use of the discussed OITs of outcome information in general (0 = Not at all, 10 = Completely). General perceived value was assessed with the question: How valuable do you think the use of the discussed OITs of outcome information is in general (0 = Not valuable at all, 10 = Very valuable)? A worse understanding was associated with more difficulty completing or understanding questionnaires, extended follow-up, and being assigned to the finger track, while patients scheduled for surgical treatment had a better understanding compared to patients scheduled for non-surgical treatment (**a**). Less perceived value was associated more difficulty completing or understanding questionnaires and being assigned to the wrist track. More perceived value was associated with getting help completing questionnaires or reading letters and a better hand function (**b**)

As the median score on general understanding was 9 [IQR 8–10], we classified patients scoring below nine as having “worse understanding” and patients scoring a nine or higher as having “better understanding”. This resulted in 1352 patients (46%) with worse understanding and 1607 patients (54%) with better understanding. Demographic and treatment factors alone (Step 1) provided an AUC of 0.57 (95%-CI 0.55–0.59). Adding mental health (Step 2) resulted in an AUC of 0.59 [0.57–0.61], while adding pain and function (Step 3) did not change the AUC. The final model, including socioeconomic factors (Step 4), yielded an AUC of 0.79 [0.77–0.80], indicating a good ability of the model to discriminate between patients with sufficient and insufficient understanding (Supplementary Table 1A). The following variables were independently associated with worse general understanding: more difficulty completing or understanding questionnaires (standardized odds ratio (SOR) 0.34 [95%-CI 031–0.38], p < 0.001), having a finger condition (0.72 [0.57–0.92], p = 0.008), longer follow-up duration (0.75 [0.61–0.91], p = 0.003) (Fig. 4a). Undergoing surgical treatment compared to non-surgical treatment (1.33 [1.11–1.59], p = 0.002) was associated with better general understanding.

#### Perceived value

The median score on general perceived value was 8 [IQR 7–10]. Thus, patients scoring below an eight were dichotomized as seeing “less perceived value” and patients scoring an eight or higher as seeing “more perceived value”. This resulted in 1091 patients (37%) with less perceived value and 1868 patients (63%) with more perceived value. Demographic and treatment factors (Step 1) provided an AUC of 0.55 (0.53–0.57). Adding mental health (Step 2) resulted in an AUC of 0.56 [0.54–0.59], and adding pain and function (Step 3) yielded an AUC of 0.57 [0.55–0.59]. The final model, including socioeconomic factors (Step 4), had an AUC of 0.75 [0.74–0.77], indicating good discriminative ability between patients perceiving less value and patients perceiving more value (Supplementary Table 1B).

Difficulty with completing or understanding questionnaires (SOR 0.40 [0.36–0.44], p < 0.001) and having a wrist condition (0.71 [0.54–0.92], p = 0.009) were associated with less perceived value while getting help with completing questionnaires or reading (1.65 [1.33–2.05], p < 0.001) and better hand function (1.12 [1.02–1.22], p = 0.021) were associated with more perceived value (Fig. 4b).

The correlation between the responses for difficulty completing or understanding questionnaires and getting help completing questionnaires was − 0.10 [95%-CI − 0.15 to − 0.05], indicating a very small correlation. This suggests these two aspects might be relatively unrelated in our sample.

## Discussion

We evaluated patient perspectives on using outcome information supported by digital OITs in clinical care using interviews and surveys, and assessed which factors were associated with general understanding and perceived value of using outcome information. Patients found using outcome information valuable and easy to understand, resulting in a positive experience with using outcome information in daily care. Using outcome information enhanced patients’ perceived understanding of symptom progression, treatment choices, and expected recovery, enabling active engagement in healthcare decisions. Outcome information empowers patients in their decision making and increases their control over choosing the most suitable treatment option for their current circumstances. The factors associated with general understanding and perceived value we identified suggest that clinicians may adapt their use of outcome information based on specific patient characteristics.

Our findings are supported by other research on the use of outcome information in daily clinical care [5, 11–15, 40–42]. Previous research demonstrates patients’ appreciation for decision-support tools using outcome information [12, 40, 41]. Graupner and colleagues found that using outcome information yields higher patient satisfaction and patient-clinician communication in patients with cancer [40]. Previous studies also suggests using outcome information can increase a patients quality of life [2, 43, 44]. Van der Willik and colleagues found that using outcome information stimulates patients to provide information they otherwise would not share with their clinicians [42]. This was confirmed by several patients during our interviews. Our study thus contributes to the increasing body of evidence that supports the use of outcome information to increase patient value, quality of life, and empowerment.

A general concern with using PROMs and the resulting outcome information in daily care is that it potentially harms the quality of care for patients with limited health literacy [13, 45, 46]. These patients might struggle with completing questionnaires and understanding the presented information. However, such OITs aim to aid patient understanding by providing visually appealing insights into their data that are easy to interpret. Although our OITs were originally developed for use by clinicians, the interviews confirmed that patients appreciated the visual presentation and that they believed it would increase their understanding of the information provided.

That patients appreciate digital OITs of outcome information, is confirmed by our finding that patients indicated they also would like to have access to their outcome information. This would enable them to monitor their progress and stay motivated to complete their treatment. Previous research also indicates that feedback on outcome information to patients resulted in higher patient satisfaction and better patient-clinician communication [40]. Therefore, future studies may explore user-friendly patient dashboards with clear presentations of the information that is easy to understand without the clinician’s explanation.

Our findings on patients with limited health literacy were somewhat inconsistent. The results of the interviews indicated that patients with limited health literacy valued the use of outcome information as they believed it can improve their understanding of the information provided. Previous studies also demonstrated that patients with limited (health) literacy appreciate the use of outcome information, especially if the information is visualized in graphs or figures, as this supports their conversation with their clinician [13, 40, 45]. However, our hierarchical regression analysis indicated that having difficulty completing or understanding questionnaires was associated with a worse general understanding and less perceived value, while patients getting help completing questionnaires or reading perceived more value in using outcome information. This may be because patients receiving help achieve a higher understanding of the data, which in turn may also create more value. This theory is supported by the very small correlation between difficulty completing or understanding questionnaires and getting help completing questionnaires. Thus, future research may identify specific strategies that help patients having difficulty completing or understanding questionnaires with these tasks to improve their general understanding and value.

The use of digital OITs such as ours may result in clinicians spending more time looking at their computers. This may be perceived as impersonal, potentially harming the patient-clinician relationship. Interestingly, our results indicate that patients believe using outcome information supported with these OITs actually enhances the patients’ interaction and communication with their clinicians, as it helps them understand the provided information, makes them feel genuinely listened to, and can serve as a platform to address sensitive topics.

This study has several limitations. One limitation is the distribution of the participants across different settings and organizations. We would have preferred to include an equal number of patients from each patient population. However, due to logistic reasons, it was impossible to include more patients from OLVG and Rijndam. Thus, our findings mainly apply to patients with hand and wrist conditions treated at a specialized treatment center. Furthermore, there is a possibility that patients who dislike or do not see any value in completing questionnaires did not complete our survey. As a result, our findings might be a relatively positive representation of reality. Furthermore, patients with limited (health) literacy might be less inclined to complete questionnaires. However, our results indicate we reached patients with both positive and negative perspectives with our survey. Additionally, in our interviews, we specifically aimed for a heterogeneous sample to also include patients with less positive experiences and patients with limited (health) literacy. Lastly, we dichotomized our data on the two general questions to perform a logistic regression, which generally reduces the richness of data. However, it was not possible to achieve reliable and clinically meaningful results using other regression methods because of the large ceiling effects in our data. Therefore, our logistic regression has value as it allowed us to provide information on factors explaining more or less perceived value and better or worse understanding.

## Conclusion

Patients have positive perspectives on using outcome information in daily care. They indicated that using outcome information supported with the digital OIT is valuable and easy to understand. Patients state that the use of outcome information by clinicians makes them feel heard. In addition, using outcome information can empower and motivate them, as it provides insights into their symptom progression and health status, making them more informed and involved in decision-making. This could potentially contribute to better VBHC. We, therefore, urge clinicians to use outcome information in their daily practice, preferably with the support of OITs, because this might improve patient independence, empowerment, and involvement in decision-making.

## Supplementary Information

Below is the link to the electronic supplementary material.

Supplementary file1 (DOCX 1241 kb)

## Data Availability

Dataset is afvailable upon request through: 10.1007/s11136-024-03685-1.
