# Correction: What are the perspectives of patients with hand and wrist conditions, chronic pain, and patients recovering from stroke on the use of patient and outcome information in everyday care? A Mixed-Methods study

**DOI:** 10.1007/s11136-024-03724-x

**Published:** 2024-07-27

**Authors:** Grada R. Arends, Nina L. Loos, Yara E. van Kooij, Kasia Tabeau, Willemijn A. de Ridder, Ruud W. Selles, Joris Veltkamp, Outcome-Based Healthcare Research Group, Robbert M. Wouters

**Affiliations:** 1 Department of Rehabilitation Medicine, Erasmus MC, Rotterdam, The Netherlands; 2 Department of Plastic, Reconstructive, and Hand Surgery, Erasmus MC, Room EE-1589, PO Box 2040, 3000 CA Rotterdam, The Netherlands; 3 Xpert Handtherapie, Utrecht, The Netherlands; 4 Erasmus School of Health Policy and Management, Erasmus University Rotterdam, Rotterdam, The Netherlands

**Correction to: Quality of Life Research** 10.1007/s11136-024-03685-1

In the original published article, the bottom part of Fig. 2 was missing. Also in the supplementary tables, the last sentence is “A negative standardized odds ratio (SOR) indicates worse understanding and positive SOR indicates better understanding.” and “A negative standardized odds ratio (SOR) indicates less perceived value and positive SOR indicates more perceived value.” However, this should be:

- Odds ratios smaller than 1.0 indicate patients have a worse understanding of the outcome information and odds ratios larger than 1.0 indicate patients have a better understanding of the outcome information. (Supplementary Table 1A)
- Odds ratios smaller than 1.0 indicate patients find outcome information less valuable and odds ratios larger than 1.0 indicate patients find outcome information more valuable (Supplementary Table 1B)

The correct version of Fig. 2 and the Supplementary information file are provided in this correction.

**Fig. 2:**
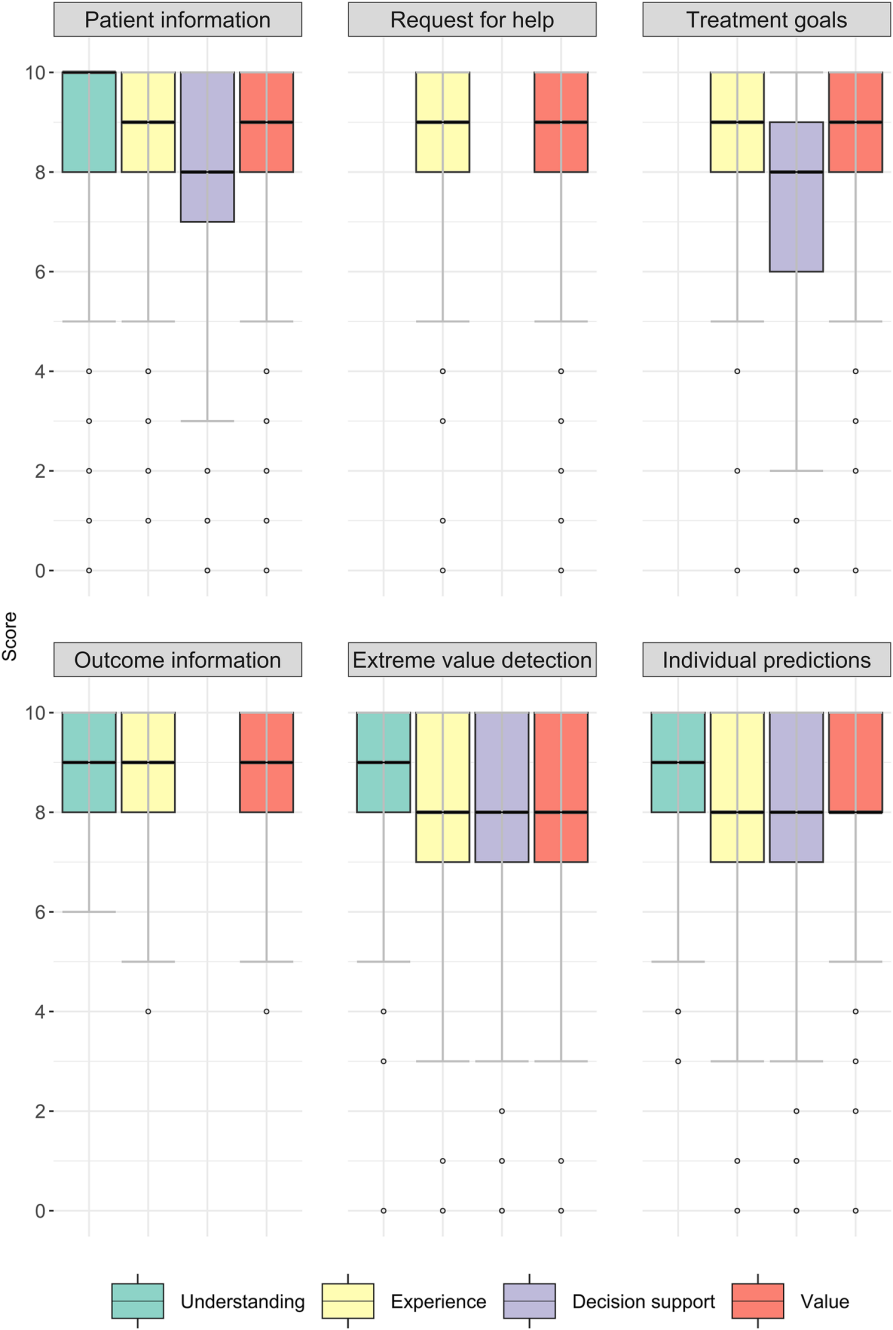
Patients' perspectives on the use of outcome information (n = 3379). In total, 3379 patients completed the survey. However, the number of patient answers differs per question because the questions about understanding, experience, decision-support, and value were only asked if the patient indicated that the OIT was discussed. The figure shows, for each OIT, the patient's answers to the questions: (1) How well did you understand the following OIT (0 = Not at all, 10 = Completely)? (2) How was your experience with the use of the following OIT (0 = Very negative, 10 = Very positive)? (3) To what extent did the following OIT support you in your decision-making about your treatment (0 = To no extent, 10 = To a big extent)? (4) How valuable do you think the use of the following OIT is (0 = Not valuable, 10 = Very valuable)?. Question 1 was not asked for the request for help and the treatment goals and Question 3 not for request for help, as patients complete these themselves. Question 3 was also not asked for the outcome information, as this information is only available after the start of the treatment and, therefore, cannot support the decision-making on the initial treatment choice

The original article has been corrected.

## Supplementary Information

Below is the link to the electronic supplementary material.

Supplementary file1 (DOCX 1243 KB)

