# Supplementary material for: What are the perspectives of patients with hand and wrist conditions, chronic pain, and patients recovering from stroke on the use of patient and outcome information in everyday care? A Mixed-Methods study
Source: Qual Life Res. 2024 Jun 5;33(9):2573–87. doi: 10.1007/s11136-024-03685-1 (PMC11390777; doi:10.1007/s11136-024-03685-1)
Supplement: Supplementary file 1 — Supplementary file1 (DOCX 1241 kb) [file 11136_2024_3685_MOESM1_ESM.docx]

***Supplementary Figures 1A.*** *The dashboard at Xpert Clinics Hand and Wrist care with the developed OITs. The figure shows the personal request for help and individual treatment goals (top left), the visuals of patient information (top left), visuals of outcome information (mid), and mental health screener (down left). Extreme values are presented in red, orange and green color codes.*

*
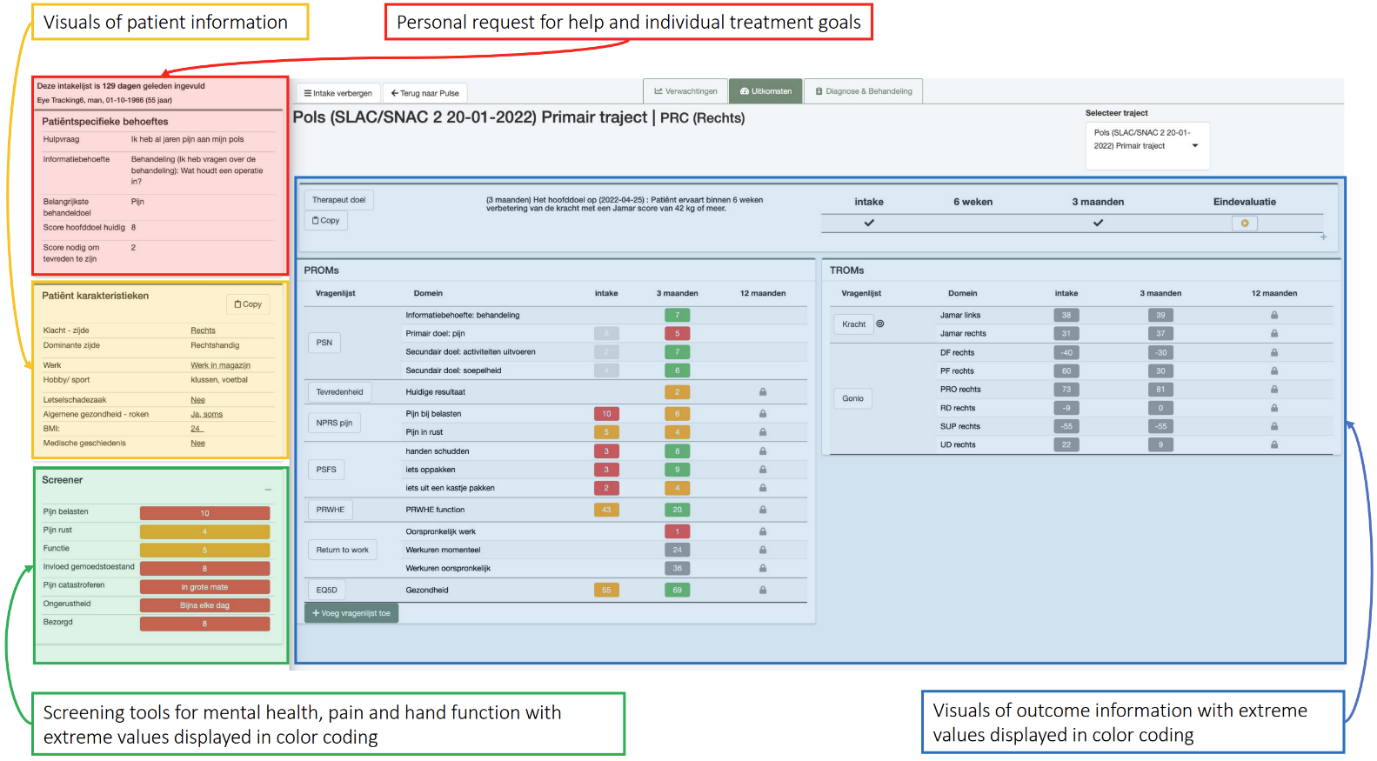
*

***Supplementary Figures 1B.*** *The dashboard at Xpert Clinics Hand and Wrist care with the developed OITs. The figure shows the individual predictions of recovery and treatment effect are displayed.*


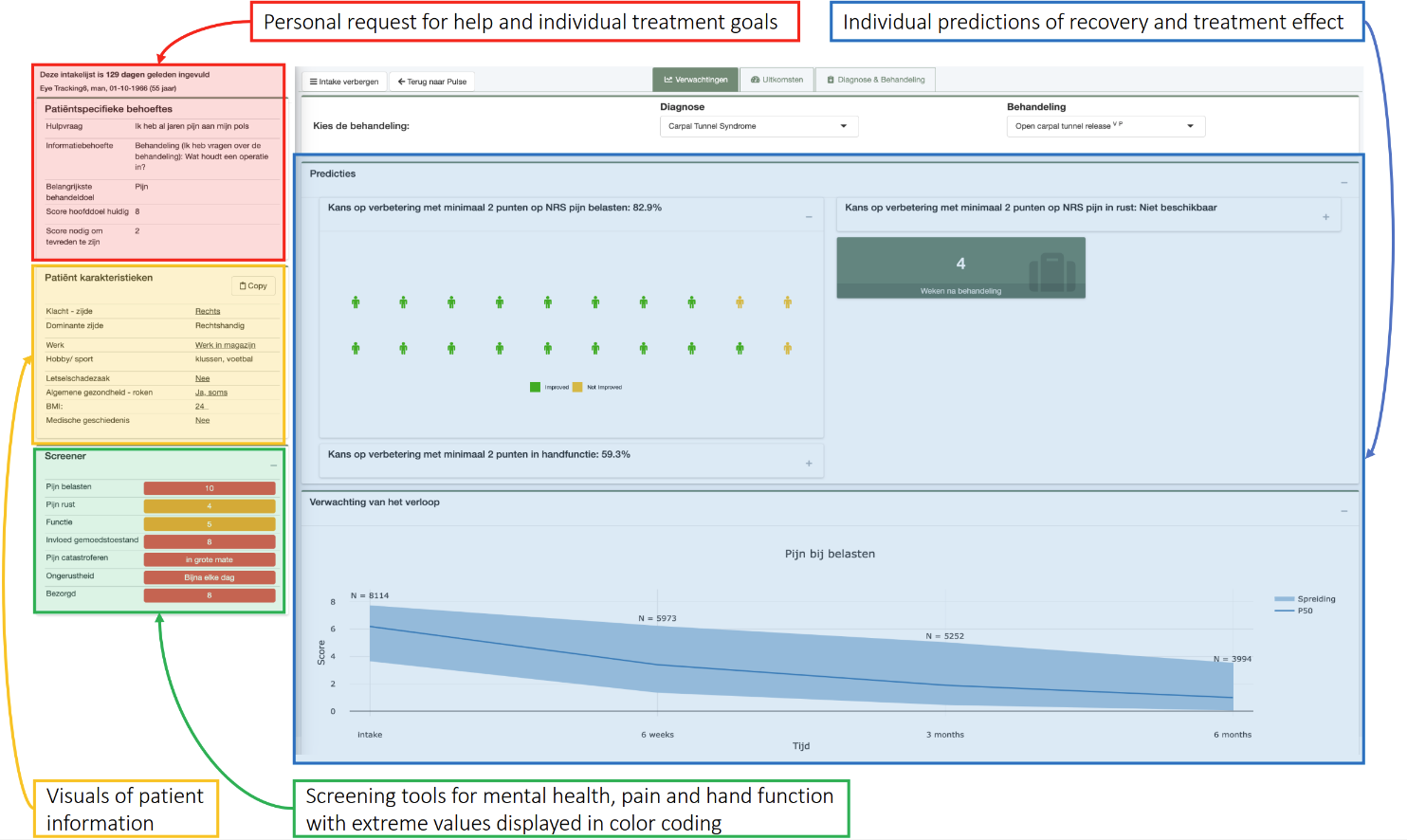


**Supplementary Figure 1C.** *The dashboard at Rijndam Revalidatie with the developed OITs.* **
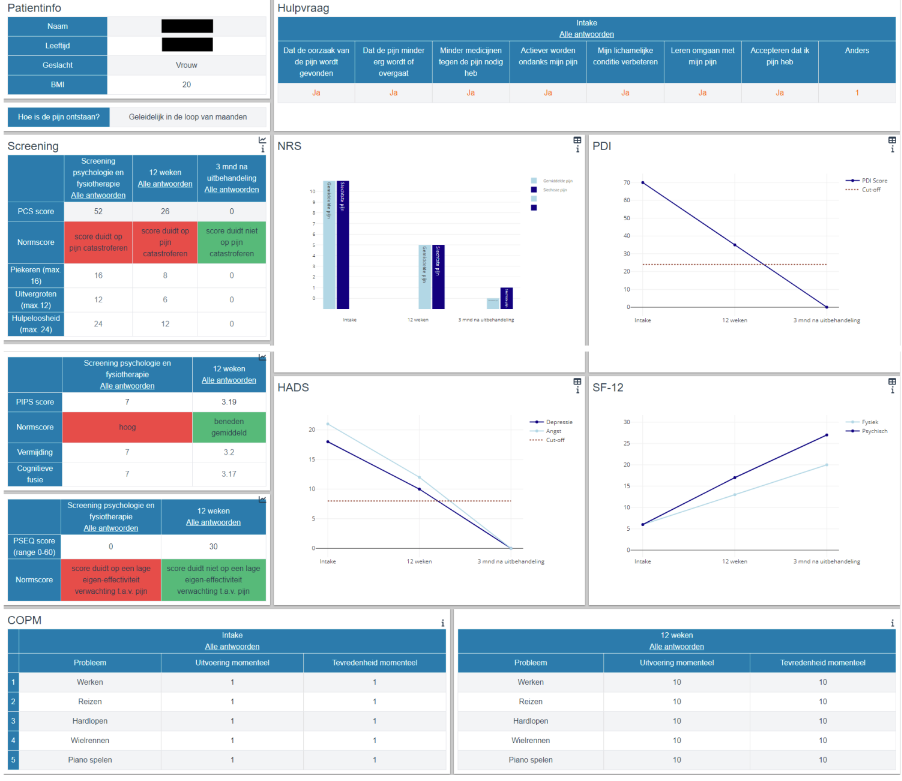
**

**
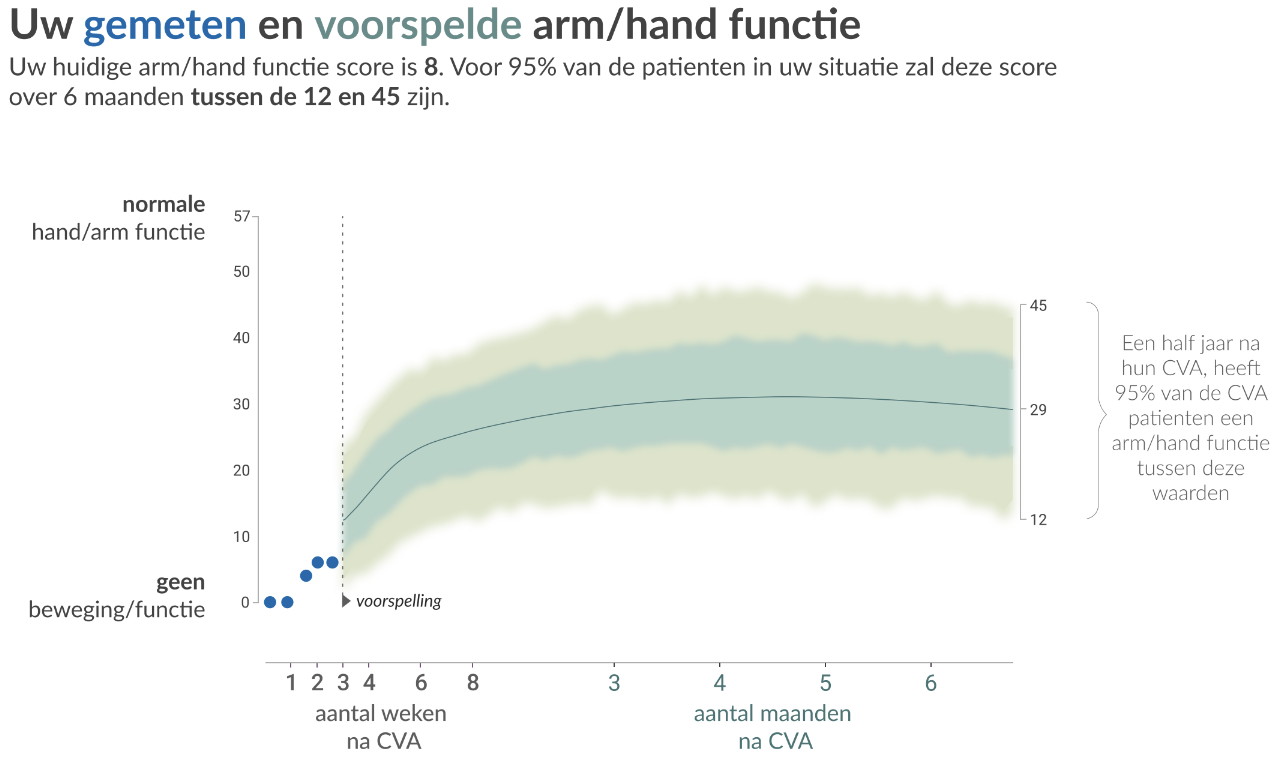
Supplementary Figure 1D.** *The prediction model at OLVG.*


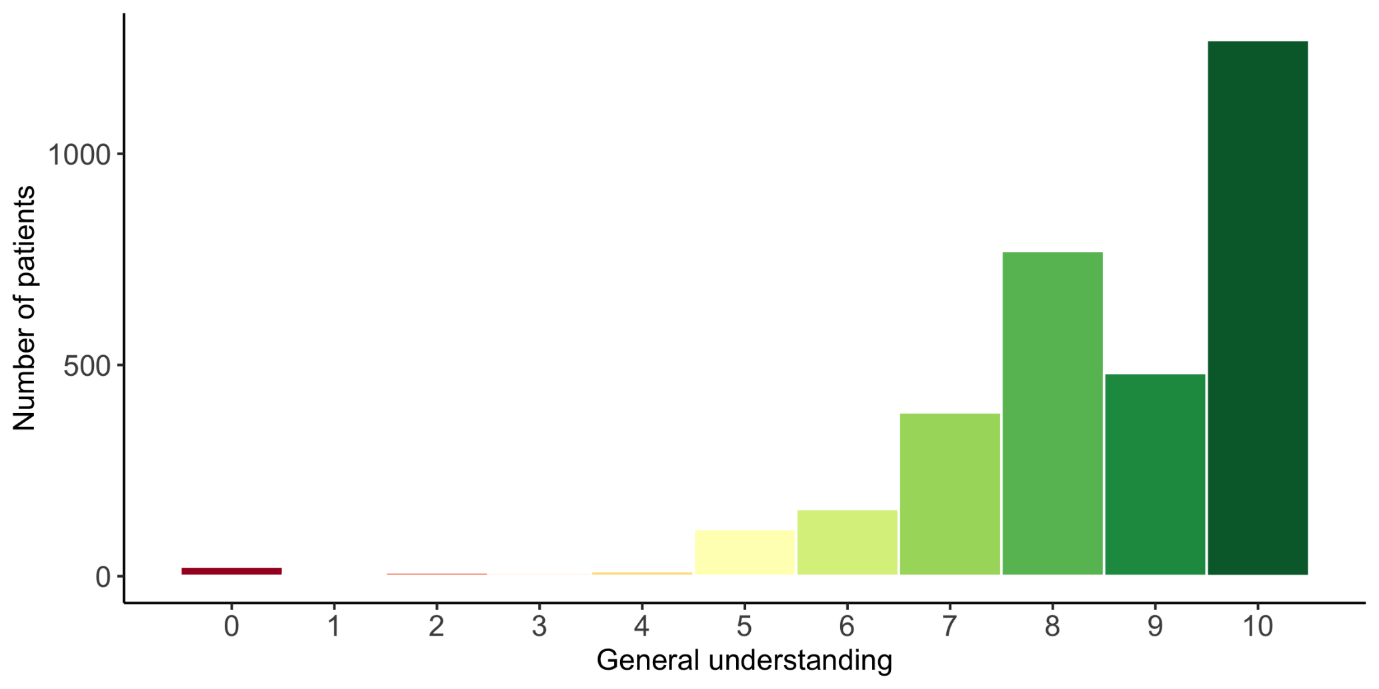
***Supplementary Figure 2A & 2B.*** *Distributions of patients’ answers on the two general questions on understanding (Supplementary Figure 2A) and perceived value (Supplementary Figure 2B) (N = 2959). Both distributions show strong ceiling effects.*


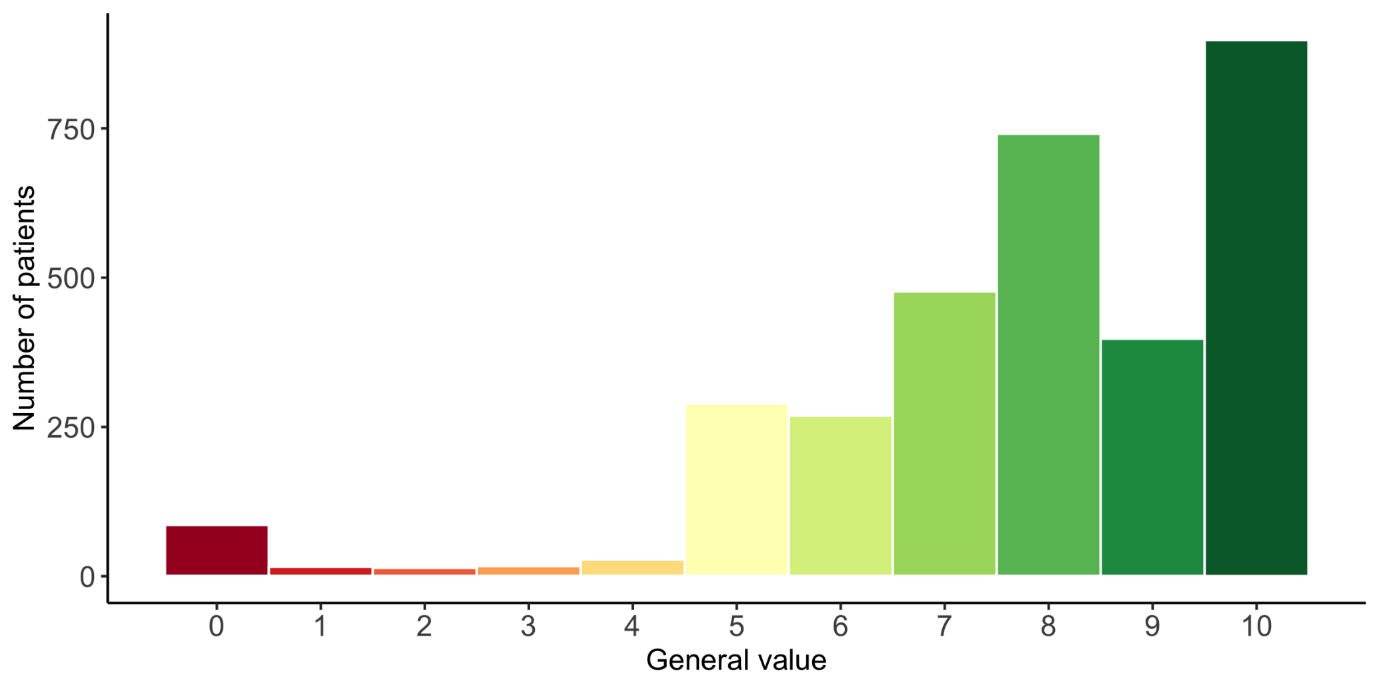


***Supplementary Table 1A.*** *Standardized odds ratios of the hierarchical logistic regression analysis for patients’ general understanding of the use of outcome information. The general understanding was measured with the question “In general, how well did you understand the use of the applications of outcome information by your doctor?” [0 = Not at all, 10 = Completely]. Odds ratios smaller than 1.0 indicate patients have a worse understanding of the outcome information and odds ratios larger than 1.0 indicate patients have a better understanding of the outcome information.*

|  | **General understanding** | | | | | | | | | |
| --- | --- | --- | --- | --- | --- | --- | --- | --- | --- | --- |
|  | **Univariable** | | **Step 1** | | **Step 2** | | **Step 3** | | **Step 4** | |
|  | **SOR [95% CI]** | **p-value** | **SOR [95% CI]** | **p-value** | **SOR [95% CI]** | **p-value** | **SOR [95% CI]** | **p-value** | **SOR [95% CI]** | **p-value** |
| Age in years | 0.98 [0.91; 1.05] | 0.597 | 0.99 [0.92; 1.07] | 0.838 | 0.99 [0.91; 1.07] | 0.782 | 1.00 [0.93; 1.09] | 0.921 | 1.07 [0.98; 1.17] | 0.143 |
| Gender: male | 0.86 [0.74; 1.00] | 0.050 | 0.85 [0.73; 0.99] | 0.038 | 0.85 [0.72; 0.99] | 0.036 | 0.85 [0.73; 1.00] | 0.047 | 0.85 [0.71; 1.01] | 0.059 |
| Second opinion: yes | 0.76 [0.60; 0.95] | 0.018 | 0.75 [0.60; 0.95] | 0.017 | 0.80 [0.63; 1.02] | 0.071 | 0.80 [0.63; 1.02] | 0.072 | 0.76 [0.58; 0.98] | 0.035 |
| Symptom duration in months | 1.00 [0.93; 1.08] | 0.939 | 1.02 [0.95; 1.10] | 0.566 | 1.02 [0.95; 1.10] | 0.612 | 1.03 [0.95; 1.11] | 0.493 | 1.05 [0.96; 1.14] | 0.293 |
| Medical history: yes | 0.92 [0.80; 1.07] | 0.286 | 0.92 [0.79; 1.07] | 0.274 | 0.92 [0.79; 1.07] | 0.288 | 0.92 [0.79; 1.07] | 0.252 | 0.97 [0.82; 1.14] | 0.686 |
| Dominant hand affected: no | 1.02 [0.87; 1.19] | 0.822 | 1.00 [0.86; 1.18] | 0.952 | 0.98 [0.84; 1.16] | 0.852 | 0.99 [0.84; 1.16] | 0.886 | 1.03 [0.86; 1.23] | 0.727 |
| Track: wrist | 0.92 [0.74; 1.14] | 0.440 | 0.82 [0.65; 1.03] | 0.089 | 0.82 [0.65; 1.04] | 0.106 | 0.83 [0.65; 1.04] | 0.107 | 0.79 [0.61; 1.03] | 0.078 |
| **Track: finger** | 0.94 [0.78; 1.14] | 0.528 | 0.81 [0.66; 1.00] | 0.051 | 0.76 [0.62; 0.94] | 0.012 | 0.78 [0.63; 0.97] | 0.025 | **0.72 [0.57; 0.92]** | **0.008** |
| Track: nerve | 1.01 [0.81; 1.27] | 0.912 | 0.77 [0.60; 1.00] | 0.046 | 0.78 [0.60; 1.01] | 0.058 | 0.82 [0.63; 1.07] | 0.140 | 0.77 [0.57; 1.03] | 0.077 |
| **Follow-up: extended** | 0.75 [0.65; 0.87] | 0.000 | 0.76 [0.64; 0.90] | 0.002 | 0.76 [0.64; 0.91] | 0.003 | 0.77 [0.64; 0.92] | 0.004 | **0.75 [0.61; 0.91]** | **0.003** |
| **Treatment type: surgical** | 1.40 [1.21; 1.63] | 0.000 | 1.40 [1.19; 1.64] | 0.000 | 1.37 [1.17; 1.61] | 0.000 | 1.40 [1.19; 1.64] | 0.000 | **1.33 [1.11; 1.59]** | **0.002** |
| Treatment type: no treatment (wait and see) | 1.24 [0.88; 1.75] | 0.212 | 1.32 [0.93; 1.87] | 0.114 | 1.30 [0.92; 1.85] | 0.138 | 1.31 [0.92; 1.86] | 0.129 | 1.40 [0.94; 2.08] | 0.096 |
| IPQ concern | 0.87 [0.80; 0.93] | 0.000 | - | - | 0.97 [0.87; 1.08] | 0.632 | 0.97 [0.87; 1.08] | 0.617 | 0.96 [0.85; 1.08] | 0.500 |
| IPQ emotional response | 0.87 [0.81; 0.93] | 0.000 | - | - | 0.93 [0.84; 1.04] | 0.221 | 0.93 [0.83; 1.03] | 0.178 | 0.99 [0.88; 1.12] | 0.918 |
| PHQ anxiety: some days | 0.72 [0.61; 0.86] | 0.000 | - | - | 0.81 [0.66; 0.99] | 0.044 | 0.82 [0.67; 1.01] | 0.063 | 0.86 [0.69; 1.08] | 0.197 |
| PHQ anxiety: more than halve of the days | 0.75 [0.57; 0.97] | 0.028 | - | - | 0.91 [0.67; 1.25] | 0.568 | 0.93 [0.68; 1.26] | 0.631 | 0.99 [0.70; 1.40] | 0.955 |
| PHQ anxiety: almost every day | 0.77 [0.62; 0.95] | 0.014 | - | - | 0.94 [0.71; 1.26] | 0.697 | 0.96 [0.72; 1.29] | 0.786 | 1.08 [0.78; 1.50] | 0.637 |
| Pain experience: to a small extent | 0.80 [0.65; 0.99] | 0.040 | - | - | 0.85 [0.68; 1.06] | 0.160 | 0.81 [0.64; 1.02] | 0.072 | 0.79 [0.61; 1.02] | 0.068 |
| Pain experience: to some extent | 0.80 [0.65; 0.99] | 0.038 | - | - | 0.91 [0.72; 1.15] | 0.427 | 0.84 [0.65; 1.09] | 0.201 | 0.87 [0.65; 1.16] | 0.352 |
| Pain experience: to a large extent | 0.75 [0.59; 0.96] | 0.022 | - | - | 0.85 [0.63; 1.14] | 0.271 | 0.78 [0.56; 1.08] | 0.130 | 0.81 [0.56; 1.16] | 0.255 |
| Pain experience: always | 0.63 [0.38; 1.05] | 0.078 | - | - | 0.73 [0.42; 1.26] | 0.259 | 0.67 [0.38; 1.19] | 0.172 | 0.60 [0.32; 1.11] | 0.105 |
| NRS hand function | 1.08 [1.00; 1.16] | 0.041 | - | - | - | - | 1.04 [0.96; 1.13] | 0.302 | 1.02 [0.93; 1.12] | 0.621 |
| NRS pain during load | 1.00 [0.93; 1.08] | 0.961 | - | - | - | - | 1.14 [1.03; 1.26] | 0.011 | 1.09 [0.97; 1.22] | 0.131 |
| NRS pain in rest | 0.95 [0.88; 1.02] | 0.155 | - | - | - | - | 0.95 [0.87; 1.05] | 0.340 | 0.94 [0.85; 1.05] | 0.290 |
| **Difficulty completing or understanding questionnaires** | 0.34 [0.31; 0.38] | 0.000 | - | - | - | - | - | - | **0.34 [0.31; 0.38]** | **0.000** |
| Getting help completing questionnaires or reading letters: yes | 0.93 [0.78; 1.12] | 0.441 | - | - | - | - | - | - | 1.11 [0.90; 1.37] | 0.310 |
| Social economic status score | 1.00 [0.93; 1.07] | 0.943 | - | - | - | - | - | - | 0.95 [0.88; 1.03] | 0.233 |
| **AUC [95% CI]** |  | | 0.57 [0.55-0.59] | | 0.59 [0.57-0.61] | | 0.59 [0.57-0.61] | | 0.79 [0.77-0.80] | |

***Supplementary Table 1B.*** *Standardized odds ratios of the hierarchical logistic regression analysis for patients’ general perceived value of the use of outcome information. The general perceived value was measured with the question “In general, how valuable do you think the use of the applications of outcome information by your doctor was?” [0 = Not valuable at all, 10 = Very valuable]. Odds ratios smaller than 1.0 indicate patients find outcome information less valuable and odds ratios larger than 1.0 indicate patients find outcome information more valuable.*

|  | **Perceived value** | | | | | | | | | |
| --- | --- | --- | --- | --- | --- | --- | --- | --- | --- | --- |
|  | **Univariable** | | **Step 1** | | **Step 2** | | **Step 3** | | **Step 4** | |
|  | **OR [95% CI]** | **p-value** | **OR [95% CI]** | **p-value** | **OR [95% CI]** | **p-value** | **OR [95% CI]** | **p-value** | **OR [95% CI]** | **p-value** |
| Age in years | 1.00 [0.93; 1.08] | 0.974 | 0.96 [0.88; 1.04] | 0.310 | 0.95 [0.87; 1.03] | 0.205 | 0.96 [0.89; 1.05] | 0.387 | 1.01 [0.92; 1.11] | 0.792 |
| Gender: male | 0.89 [0.76; 1.04] | 0.134 | 0.88 [0.75; 1.03] | 0.117 | 0.89 [0.76; 1.04] | 0.139 | 0.90 [0.76; 1.06] | 0.191 | 0.90 [0.76; 1.07] | 0.242 |
| Second opinion: yes | 0.80 [0.63; 1.01] | 0.057 | 0.81 [0.64; 1.03] | 0.083 | 0.83 [0.66; 1.06] | 0.139 | 0.84 [0.66; 1.07] | 0.162 | 0.78 [0.60; 1.01] | 0.063 |
| Symptom duration in months | 0.98 [0.91; 1.05] | 0.528 | 0.97 [0.90; 1.05] | 0.417 | 0.97 [0.90; 1.05] | 0.415 | 0.97 [0.90; 1.05] | 0.515 | 0.99 [0.91; 1.08] | 0.788 |
| Medical history: yes | 1.01 [0.87; 1.17] | 0.898 | 1.00 [0.85; 1.16] | 0.964 | 1.00 [0.85; 1.17] | 0.982 | 0.99 [0.85; 1.16] | 0.934 | 1.03 [0.86; 1.22] | 0.768 |
| Dominant hand affected: no | 1.07 [0.91; 1.25] | 0.431 | 1.07 [0.90; 1.26] | 0.444 | 1.06 [0.90; 1.26] | 0.462 | 1.07 [0.91; 1.27] | 0.412 | 1.12 [0.94; 1.35] | 0.201 |
| **Track: wrist** | 0.74 [0.59; 0.92] | 0.008 | 0.73 [0.58; 0.93] | 0.011 | 0.74 [0.58; 0.94] | 0.015 | 0.74 [0.59; 0.95] | 0.016 | **0.71 [0.54; 0.92]** | **0.009** |
| Track: finger | 0.88 [0.72; 1.07] | 0.205 | 0.89 [0.72; 1.10] | 0.281 | 0.90 [0.72; 1.12] | 0.329 | 0.92 [0.74; 1.15] | 0.454 | 0.88 [0.69; 1.12] | 0.297 |
| Track: nerve | 0.86 [0.68; 1.09] | 0.215 | 0.88 [0.67; 1.15] | 0.339 | 0.90 [0.69; 1.18] | 0.454 | 0.93 [0.71; 1.21] | 0.574 | 0.89 [0.66; 1.20] | 0.437 |
| Follow-up: extended | 1.04 [0.90; 1.22] | 0.579 | 1.09 [0.91; 1.31] | 0.328 | 1.12 [0.93; 1.34] | 0.218 | 1.13 [0.94; 1.36] | 0.181 | 1.19 [0.98; 1.45] | 0.081 |
| Treatment type: surgical | 1.17 [1.00; 1.36] | 0.050 | 1.23 [1.05; 1.45] | 0.012 | 1.22 [1.04; 1.44] | 0.017 | 1.24 [1.06; 1.47] | 0.009 | 1.16 [0.97; 1.38] | 0.114 |
| Treatment type: no treatment (wait and see) | 0.75 [0.53; 1.05] | 0.097 | 0.79 [0.56; 1.12] | 0.195 | 0.81 [0.57; 1.15] | 0.248 | 0.81 [0.57; 1.15] | 0.242 | 0.78 [0.53; 1.15] | 0.216 |
| IPQ concern | 0.93 [0.86; 1.00] | 0.060 | - | - | 0.96 [0.86; 1.07] | 0.465 | 0.96 [0.86; 1.07] | 0.477 | 0.95 [0.84; 1.07] | 0.374 |
| IPQ emotional response | 0.91 [0.84; 0.98] | 0.011 | - | - | 0.87 [0.78; 0.98] | 0.019 | 0.87 [0.77; 0.97] | 0.013 | 0.90 [0.79; 1.02] | 0.092 |
| PHQ anxiety: some days | 0.99 [0.84; 1.18] | 0.950 | - | - | 1.13 [0.92; 1.40] | 0.239 | 1.16 [0.94; 1.43] | 0.172 | 1.28 [1.02; 1.61] | 0.037 |
| PHQ anxiety: more than halve of the days | 1.09 [0.83; 1.43] | 0.532 | - | - | 1.30 [0.94; 1.80] | 0.106 | 1.33 [0.96; 1.84] | 0.082 | 1.46 [1.03; 2.08] | 0.035 |
| PHQ anxiety: almost every day | 0.94 [0.76; 1.17] | 0.573 | - | - | 1.18 [0.88; 1.59] | 0.272 | 1.21 [0.90; 1.63] | 0.209 | 1.37 [0.99; 1.90] | 0.060 |
| Pain experience: to a small extent | 1.10 [0.88; 1.36] | 0.408 | - | - | 1.15 [0.91; 1.44] | 0.235 | 1.10 [0.86; 1.40] | 0.444 | 1.12 [0.86; 1.45] | 0.408 |
| Pain experience: to some extent | 1.04 [0.84; 1.29] | 0.699 | - | - | 1.15 [0.90; 1.45] | 0.266 | 1.08 [0.83; 1.41] | 0.568 | 1.15 [0.86; 1.53] | 0.347 |
| Pain experience: to a large extent | 1.14 [0.88; 1.47] | 0.325 | - | - | 1.29 [0.95; 1.74] | 0.101 | 1.20 [0.86; 1.68] | 0.276 | 1.36 [0.95; 1.96] | 0.095 |
| Pain experience: always | 1.11 [0.66; 1.89] | 0.688 | - | - | 1.33 [0.75; 2.35] | 0.324 | 1.23 [0.69; 2.21] | 0.486 | 1.21 [0.65; 2.27] | 0.551 |
| **NRS hand function** | 1.07 [1.00; 1.16] | 0.066 | - | - | - | - | 1.12 [1.02; 1.21] | 0.012 | **1.12 [1.02; 1.22]** | **0.021** |
| NRS pain during load | 1.06 [0.98; 1.14] | 0.130 | - | - | - | - | 1.11 [1.00; 1.24] | 0.043 | 1.06 [0.95; 1.19] | 0.296 |
| NRS pain in rest | 1.04 [0.96; 1.12] | 0.361 | - | - | - | - | 1.02 [0.92; 1.13] | 0.696 | 1.02 [0.92; 1.14] | 0.672 |
| **Difficulty completing or understanding questionnaires** | 0.41 [0.38; 0.45] | 0.000 | - | - | - | - | - | - | **0.40 [0.36; 0.44]** | **0.000** |
| **Getting help completing questionnaires or reading letters: yes** | 1.30 [1.07; 1.57] | 0.007 | - | - | - | - | - | - | **1.65 [1.33; 2.05]** | **0.000** |
| Social economic status score | 0.96 [0.89; 1.03] | 0.253 | - | - | - | - | - | - | 0.92 [0.85; 1.00] | 0.051 |
| **AUC [95% CI]** |  | | 0.55 [0.53-0.57] | | 0.56 [0.54-0.59] | | 0.57 [0.55-0.59] | | 0.75 [0.74-0.77] | |
